# Penfluridol triggers cytoprotective autophagy and cellular apoptosis through ROS induction and activation of the PP2A-modulated MAPK pathway in acute myeloid leukemia with different FLT3 statuses

**DOI:** 10.1186/s12929-019-0557-2

**Published:** 2019-08-31

**Authors:** Szu-Yuan Wu, Yu-Ching Wen, Chia-Chi Ku, Yi-Chieh Yang, Jyh-Ming Chow, Shun-Fa Yang, Wei-Jiunn Lee, Ming-Hsien Chien

**Affiliations:** 1 Department of Radiation Oncology, Wan Fang Hospital, Taipei Medical University, Taipei, Taiwan; 2 Department of Radiology, School of Medicine, College of Medicine, Taipei Medical University, Taipei, Taiwan; 3 Department of Urology, Wan Fang Hospital, Taipei Medical University, Taipei, Taiwan; 4 Department of Urology, School of Medicine, College of Medicine, Taipei Medical University, Taipei, Taiwan; 5 Graduate Institute of Clinical Medicine, College of Medicine, Taipei Medical University, Taipei, Taiwan; 6 Division of Hematology and Medical Oncology, Department of Internal Medicine, Wan Fang Hospital, Taipei Medical University, Taipei, Taiwan; 7 Institute of Medicine, Chung Shan Medical University, Taichung, Taiwan; 8 Department of Medical Research, Chung Shan Medical University Hospital, Taichung, Taiwan; 9 Department of Medical Education and Research, Wan Fang Hospital, Taipei Medical University, Taipei, Taiwan; 10 Pulmonary Research Center, Wan Fang Hospital, Taipei Medical University, Taipei, Taiwan; 11 TMU Research Center of Cancer Translational Medicine, Taipei Medical University, Taipei, Taiwan

**Keywords:** Acute myeloid leukemia, Apoptosis, Autophagy, Protein phosphatase 2 a, Akt, Mitogen-activated protein kinase, Reactive oxygen species, Penfluridol

## Abstract

**Background:**

Chemotherapy is the main treatment for acute myeloid leukemia (AML), but the cure rates for AML patients remain low, and the notorious adverse effects of chemotherapeutic drugs drastically reduce the life quality of patients. Penfluridol, a long-acting oral antipsychotic drug, has an outstanding safety record and exerts oncostatic effects on various solid tumors. Until now, the effect of penfluridol on AML remains unknown.

**Methods:**

AML cell lines harboring wild-type (WT) Fms-like tyrosine kinase 3 (FLT3) and internal tandem duplication (ITD)-mutated FLT3 were used to evaluate the cytotoxic effects of penfluridol by an MTS assay. A flow cytometric analysis and immunofluorescence staining were employed to determine the cell-death phenotype, cell cycle profile, and reactive oxygen species (ROS) and acidic vesicular organelle (AVO) formation. Western blotting and chemical inhibitors were used to explore the underlying mechanisms involved in penfluridol-mediated cell death.

**Results:**

We observed that penfluridol concentration-dependently suppressed the cell viability of AML cells with FLT3-WT (HL-60 and U937) and FLT3-ITD (MV4–11). We found that penfluridol treatment not only induced apoptosis as evidenced by increases of nuclear fragmentation, the sub-G_1_ populations, poly (ADP ribose) polymerase (PARP) cleavage, and caspase-3 activation, but also triggered autophagic responses, such as the light chain 3 (LC3) turnover and AVO formation. Interestingly, blocking autophagy by the pharmacological inhibitors, 3-methyladenine and chloroquine, dramatically enhanced penfluridol-induced apoptosis, indicating the cytoprotective role of autophagy in penfluridol-treated AML cells. Mechanistically, penfluridol-induced apoptosis occurred through activating protein phosphatase 2A (PP2A) to suppress Akt and mitogen-activated protein kinase (MAPK) activities. Moreover, penfluridol’s augmentation of intracellular ROS levels was critical for the penfluridol-induced autophagic response. In the clinic, we observed that patients with AML expressing high PP2A had favorable prognoses.

**Conclusions:**

These findings provide a rationale for penfluridol being used as a PP2A activator for AML treatment, and the combination of penfluridol with an autophagy inhibitor may be a novel strategy for AML harboring FLT3-WT and FLT3-ITD.

**Electronic supplementary material:**

The online version of this article (10.1186/s12929-019-0557-2) contains supplementary material, which is available to authorized users.

## Background

Acute myeloid leukemia (AML), the most common type of leukemia in adults, is an aggressive disease caused by the transformation of hematopoietic progenitor cells due to the acquisition of multiple genetic alterations. Although intensive chemotherapy improves outcomes for patients with AML, most eventually die of the disease and suffer significant toxicities such as anemia, bleeding, and infection due to side effects of the therapy [1]. Hence, alternative treatments with high efficacy and low toxicity urgently need to be found.

Fms-like tyrosine kinase 3 (FLT3) is a class III transmembrane receptor tyrosine kinase family that functions to induce cell proliferation and survival via activating phosphatidylinositol-3 kinase (PI3K), Akt, mitogen-activated protein kinase (MAPK), and signal transducer and activator of transcription 5 (STAT5) signaling pathways [2]. In AML cells harboring wild-type FLT3 (FLT3-WT), co-expression of FLT3 and its ligand (FL) were frequently observed, and establishing an autocrine signaling loop resulted in constitutive FLT3 signaling [3]. Moreover, about 24% of adult AML patients were observed to carry a Juxta-membrane domain internal tandem duplication (ITD) mutation in the FLT3 gene (FLT3-ITD), which leads to uncontrolled cellular proliferation and survival through constitutive activation of FLT3 and subsequent hyperactivation of its downstream signaling pathway [2, 4].

Protein phosphatase 2A (PP2A), a heterotrimeric serine/threonine phosphatase composed of structural, regulatory, and catalytic subunits in mammalian cells, is a tumor suppressor that inactivates multiple components of growth and survival signaling pathways required for tumorigenesis such as the Akt, MAPK, and Wnt signaling pathways [5–7]. PP2A inactivation frequently occurs in several solid and non-solid tumors including AML, leading to sustained activation of survival pathways or inhibition of apoptotic pathways [5, 8, 9]. PP2A is currently recognized as a druggable tumor suppressor in AML [10]. Recently, Smith et al. demonstrated that pharmacological activation of PP2A inhibited FLT3-mediated growth and survival of AML cells, and suggested that PP2A activation may be a therapeutic strategy for treating FLT3-driven malignancies [11].

Autophagy is a process whereby cells digest their own organelles in such stressful conditions as nutrient deprivation, hypoxia, or exposure to chemotherapy, and this ultimately maintains cancer cell survival [12]. In AML, the hypoxic bone marrow niche contributes to chemoresistance via upregulating an autophagic pathway potentially allowing persistence of minimal residual disease after chemotherapy and eventually leading to relapse. A recent study indicated that targeting autophagy might be a therapeutic strategy for AML [13]. A growing body of evidence indicates that apoptosis and autophagy are interdependent, and activation of these two machineries most often occurs simultaneously [14]. Understanding the interplay between apoptosis and autophagy in AML would be crucial in improving therapeutic efficiency.

During the past decade, many natural compounds with low toxicity have received much attention for their potential chemotherapeutic and chemopreventive properties against AML [15]. However, the major limitations of these compounds for clinical use are their low aqueous solubility and oral bioavailability which cause them to have low efficacy in the human body [16]. In contrast to these natural compounds, penfluridol is a long-acting oral antipsychotic drug for the clinical treatment of schizophrenia in use since 1970 [17]. Recently, repurposing of penfluridol to suppress the growth or metastasis of several solid tumor types (breast, pancreatic, and brain tumors) was demonstrated in both in vitro and in vivo models [18–24]. Apoptosis and autophagy are two major death machineries induced by penfluridol in these solid tumors. In contrast to solid tumors, the anticancer potential and underlying mechanisms of the anticancer effect of penfluridol against AML are still unknown.

In the present study, we examined the cytotoxic effects of penfluridol in three AML cancer cell lines which harbor the FLT3-WT and FLT3-ITD. We further clarified its effects on the caspase-mediated crosstalk between apoptosis and autophagy. Our findings provide new information for the potential repurposing of penfluridol for treating AML.

## Materials and methods

### Antibodies, reagents, and chemical compounds

Antibodies against LC3B, p62, the cleaved caspase-3 and poly (ADP ribose) polymerase (PARP), and unphosphorylated or phosphorylated forms of Akt, extracellular signal-regulated kinase 1/2 (ERK1/2), p38, and c-Jun N-terminal kinase 1/2 (JNK1/2) were all purchased from Cell Signaling Technology (Danvers, MA). An antibody specific for phosphorylated form of PP2A-Cα (Tyr307) was purchased from Thermo Fisher Scientific (Waltham, MA). An antibody specific for β-actin was obtained from Santa Cruz Biotechnology (Santa Cruz, CA). Penfluridol (P3371) and dimethyl sulfoxide (DMSO) were purchased from Sigma-Aldrich (St. Louis, MO). A 40-mM stock solution of penfluridol was made in DMSO and stored at − 20 °C. Chemical inhibitors or activators including cycloheximide (CHX), N-acetylcysteine (NAC), glutathione (GSH), ZVAD-FMK, 3-methylamphetamine (3-MA), chloroquine (CQ), and FTY720 were also obtained from Sigma-Aldrich. The MAPK inhibitors, U0126, SP600126, and SB203580, were purchased from Calbiochem (San Diego, CA). The PP2A inhibitor, okadaic acid (OA), was obtained from Abcam (Cambridge, MA). Fetal bovine serum (FBS), antibiotics, and other medium additives were obtained from Life Technologies (Gaithersburg, MD).

### Cell culture

The human leukemic HL-60, U937, and MV4–11 cell lines, each harboring FLT3-WT or FLT3-ITD, were obtained from ATCC (Manassas, VA). All leukemic cell lines were cultured in RPMI 1640 medium (Invitrogen, Carlsbad, CA) supplemented with 10% heat-inactivated FBS, 2 mM L-glutamine, 100 U/mL penicillin, and 100 μg/mL streptomycin. Cells were maintained at 37 °C in a humidified atmosphere of 95% air and 5% CO_2_.

### In vitro cell viability assay

AML cells at a density of 3 × 10^4^ cells/well were seeded into 24-well culture plates containing complete media and incubated for 1 day, after which they were exposed to different concentrations of penfluridol for the indicated time points, and cell viabilities were determined by an MTS assay (Promega, Madison, WI). The absorbance (A) was read at 490 nm using an enzyme-linked immunosorbent assay (ELISA) reader (MQX200; Bio-Tek Instruments, Winooski, VT). The cell viability rate (%) was analyzed by the formula: A_490, penfluridol_/A_490, vehicle_ × 100, and the half maximal inhibitory concentration (IC_50_) was further determined.

### Nuclear morphological analysis by DAPI

Apoptotic chromatin condensation in nuclei was determined by DNA staining with the fluorescent dye, 4′,6-diamidino-2-phenylindole (DAPI, Sigma). After penfluridol treatment for 24 h, cells were fixed with methanol for 10 min, stained with the DAPI solution for 5 min, and examined and photographed using a Zeiss Axiophot fluorescence microscope (Carl Zeiss Microimaging, Gottingen, Germany).

### Fluorescence-activated cell sorting (FACS) analysis of cell cycle profiles and apoptosis

AML cells (2 × 10^6^ cells/ml) were treated with 0.5% DMSO or penfluridol (5 or 7.5 μM) for 24 h. Cells were then fixed in 70% ethanol and stored at − 20 °C overnight, after which they were washed with ice-cold phosphate-buffered saline (PBS) and stained with a propidium iodide (PI) solution (4 μg/m PI, 0.5 mg/ml RNase A, and 1% Triton X-100 in PBS) for 30 min in the dark followed by filtration using a 40-μm nylon mesh (Falcon, San Jose, CA). The DNA contents were measured using a FACScan laser flow cytometer analysis system (Beckman Coulter, Los Angeles, CA). The proportion of nuclei in each phase of the cell cycle was measured, and apoptotic cells were quantified by measuring the sub-G_1_ population of the cell cycle.

### Western blotting

Total cell lysate extraction and protein contents were respectively prepared and determined as previously described [25]. Equal amounts of protein extracts (20~30 μg) were separated by sodium dodecylsulfate polyacrylamide gel electrophoresis (SDS-PAGE), transferred onto polyvinylidene difluoride (PVDF) membranes (Bio-Rad, Hercules, CA), and probed with indicated primary antibodies and horseradish peroxidase (HRP)-conjugated secondary antibodies. After washing, blots were incubated with enhanced chemiluminescence reagent (Amersham, Arlington Heights, IL) and signals were detected by the chemiluminescence imaging system, MultiGel-21, (TOP BIO, New Taipei City, Taiwan). Densitometric analysis of the bands was carried out using Image-Pro Plus software (Media Cybernetics, Rockville, MD).

### Measurement of reactive oxygen species (ROS) production

AML cells were treated with penfluridol or H_2_O_2_ for the indicated time points, and the intracellular ROS production was detected by staining with the fluoroprobe, 2′,7′-dichlorofluorescin diacetate (DCFDA, Sigma), in RPMI 1640 medium for 30 min. After washing cells with PBS, ROS production of DCFDA-preloaded cells was captured using a fluorescence microscope (Zeiss Axioplan) or quantified by flow cytometry.

### Detection and quantification of autophagic cells by staining with acridine orange (AO)

Formation of acidic vesicular organelles (AVOs), a morphological characteristic of autophagy, was detected by AO (Sigma) staining. AML cells were treated with 7.5 μM penfluridol for 12 h. Autophagic vacuoles were labeled with AO by incubating cells with 1 μg/ml AO in PBS at 37 °C for 20 min. After incubation, cells were washed with PBS, fresh phenol red-free media was added, and the number of cells with increased AVOs was determined by flow cytometry. At least 20,000 cells within the gated region were analyzed.

### Statistical analysis

Data are expressed as the mean ± standard deviation (SD). Statistical differences were determined using the Statistical Package for Social Science software, vers. 16 (SPSS, Chicago, IL). Data were analyzed using Student’s *t*-test with *p* < 0.05 as the criterion of significance when two groups were compared.

## Results

### Penfluridol reduces cell viability of human AML cells harboring FLT3-WT or the FLT3-ITD mutation

**Fig. 1:**
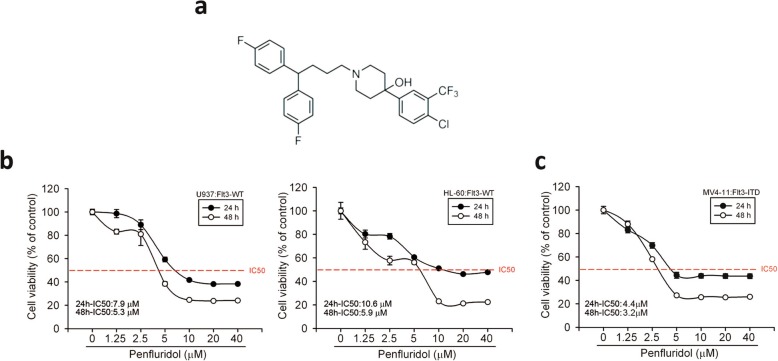
Cytotoxicity of penfluridol in human acute myeloid leukemia (AML) cells harboring different Fms-like tyrosine kinase 3 (FLT3) statuses. **a** The chemical structure of penfluridol. **b** and **c** Three AML cell lines including HL-60 and U937 cells (wild-type FLT3), and MV4–11 (mutant FLT3, internal tandem duplication), were treated with vehicle or indicated concentrations of penfluridol for 24 and 48 h, respectively. Cell viability was measured using an MTS assay. Values are presented as the percentage of cell inhibition, where vehicle-treated cells were regarded as 100%. Data are presented as the mean ± SD of three independent experiments. The 50% growth inhibitory concentration (IC_50_) values after 24- and 48-h treatment with penfluridol in each cell line are shown

The chemical structure of penfluridol is shown in Fig. 1a. To determine the pharmacological potential of penfluridol against AML cells, we investigated the effects of various concentrations (1.25~40 μM) of penfluridol on the growth of three human AML cell lines, HL-60 (FLT3-WT), U937 (FLT3-WT), and MV4–11 (FLT3-ITD), for 24 and 48 h using an MTS assay. As shown in Fig. 1b and c, penfluridol significantly reduced cell viability in a concentration-dependent manner, and IC_50_ values were determined for the three AML cell lines. In these tested AML cell lines, MV4–11 cells were the most sensitive to penfluridol treatment for 24 h (IC_50_: 4.4 μM) and 48 h (IC_50_: 3.2 μM) (Fig. 1c).

### Penfluridol treatment results in the apoptosis of AML cells harboring FLT3-WT and FLT3-ITD mutation

**Fig. 2:**
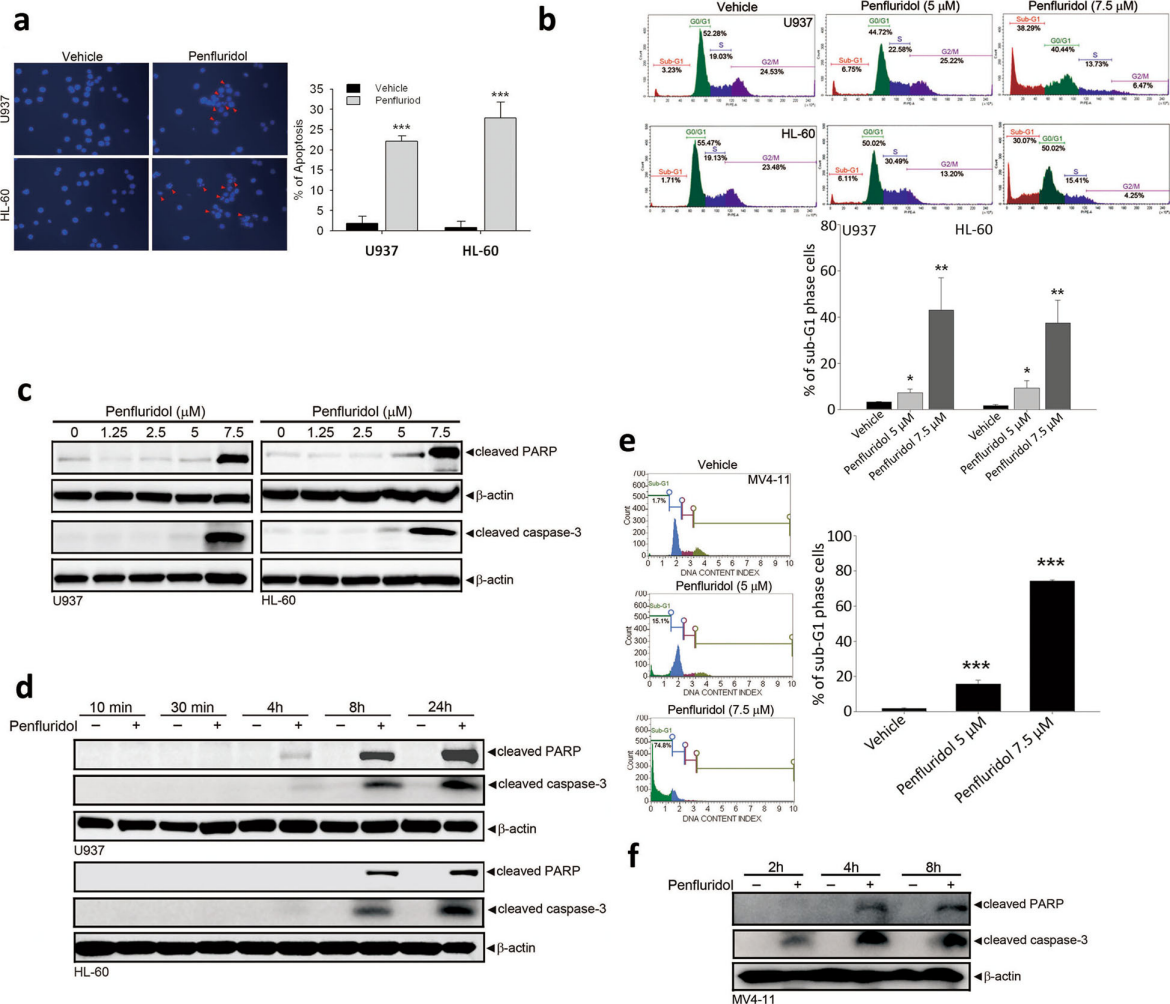
Penfluridol induces apoptosis of human acute myeloid leukemia (AML) cells with wild-type or mutant Fms-like tyrosine kinase 3 (FLT3). **a** HL-60 and U937 cells with wild-type FLT3 were treated with 7.5 μM penfluridol for 24 h, and nuclear fragmentation and condensation (arrows) as indicators of apoptosis were analyzed by fluorescence microscopy after DAPI staining, and the percentage of apoptotic cells was determined. Data are presented as the mean ± SD. *** *p* < 0.001 versus the control group. **b** HL-60 and U937 cells were treated with 7.5 μM penfluridol for 24 h and stained with propidium iodide (PI), then analyzed by flow cytometry. Percentages of cells at the G_0_/G_1_, S, G_2_/M, and sub-G_1_ phases were determined. Data are presented as the mean ± SD. * *p* < 0.05; ** *p* < 0.01 versus the control group. **c** and **d** HL-60 and U937 cells were treated with different concentrations of penfluridol for 24 h (**c**) or 7.5 μM penfluridol for different time points (**d**), and then the apoptosis-associated proteins, cleaved poly (ADP ribose) polymerase (PARP) and caspase-3, were detected by a Western blot analysis. β-actin served as a loading control. **e** and **f** Cell death in the sub-G_1_ phase (**e**) and cleaved PARP and caspase-3 proteins (**f**) were increased after penfluridol treatment with different concentrations and at different time points, in MV4–11 cells harboring mutant FLT3 (internal tandem duplication). Data are presented as the mean ± SD. *** *p* < 0.001 versus the control group

To investigate the mode of cell death induced by penfluridol, HL-60 and U937 cells harboring FLT3-WT were treated with penfluridol (7.5 μM) for 24 h and stained with DAPI. Both penfluridol-treated cell lines exhibited typical morphological changes due to cellular apoptosis, such as condensed nuclear chromatin and nuclear fragmentation (Fig. 2a, red arrows). To further confirm that cellular apoptosis was induced by penfluridol, a FACS analysis was performed. As shown in Fig. 2b, penfluridol induced concentration-dependent increases in the sub-G_1_ population in both U937 and HL-60 cells. Caspase-3 activation-mediated PARP cleavage is a hallmark of apoptosis. Our results showed that caspase-3 activation and corresponding PARP-1 cleavage were triggered by penfluridol in concentration- (Fig. 2c) and time-dependent (Fig. 2d) manners. In addition to FLT3-WT AML cells, the apoptosis-inducing effect of penfluridol was more dominant in MV4–11 cells with FLT3-ITD than HL-60 or U937 cells as evidenced by the higher sub-G_1_ cell accumulation induced by penfluridol (Fig. 2e). Moreover, penfluridol also time-dependently induced caspase-3 activation and PARP cleavage in MV4–11 cells (Fig. 2f). Taken together, the results indicate that penfluridol might be useful as a therapeutic agent in managing AML cells with different FLT3 statuses.

### Penfluridol triggers an apoptotic effect via inducing PP2A activation and further modulating MAPK pathways

**Fig. 3:**
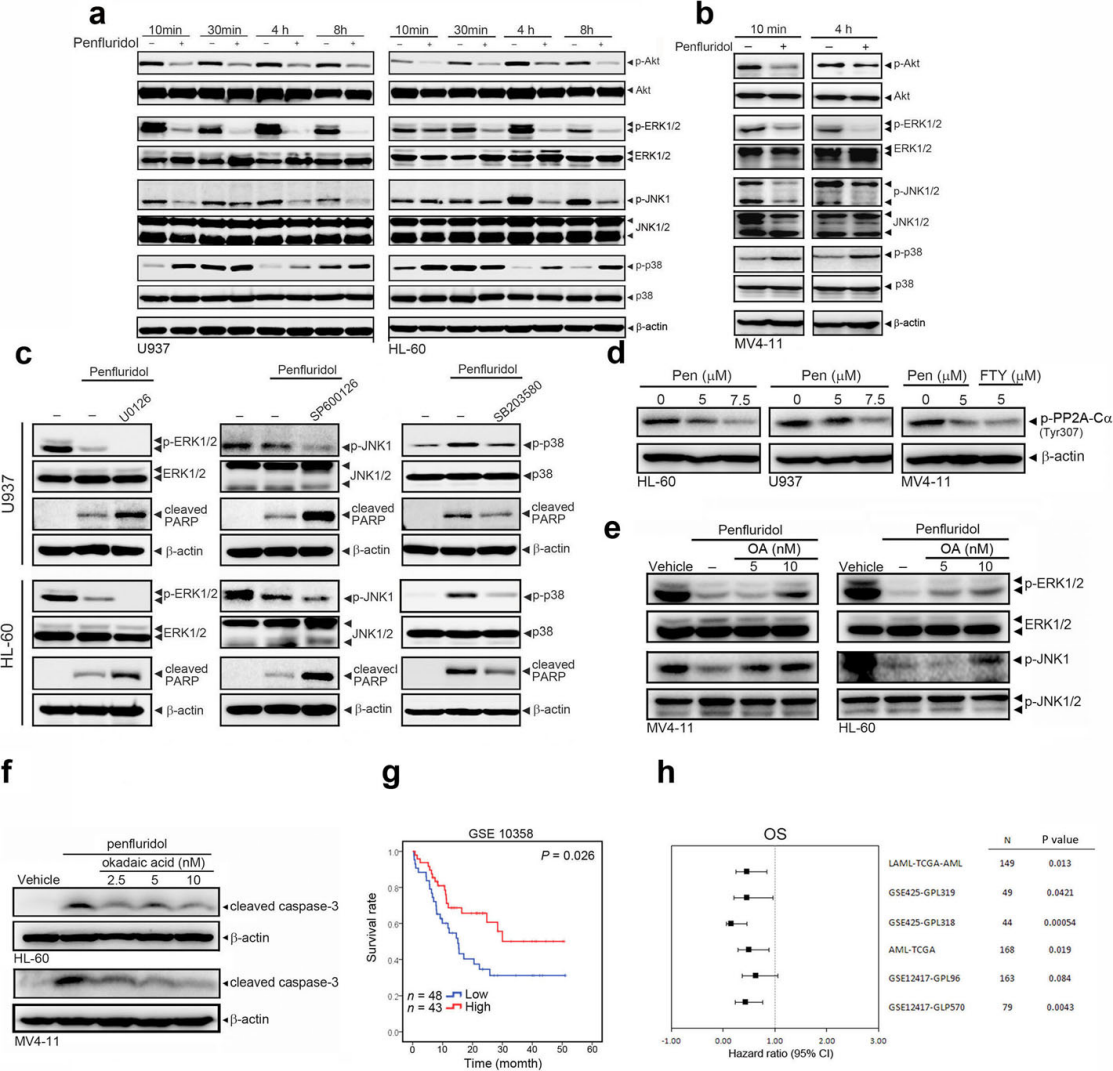
Penfluridol triggers an apoptotic effect via induction of the protein phosphatase 2A (PP2A)-modulated deactivation of mitogen-activated protein kinase (MAPK) pathways. **a** and **b** Phosphorylation levels of Akt, ERK1/2, JNK1/2, and p38 were assessed using a Western blot analysis after treatment of Fms-like tyrosine kinase 3 (FLT3)-wild type (WT) acute myeloid leukemia (AML) cells, U937 and HL-60, (**a**) or FLT3 mutant (internal tandem duplication) cells, MV4–11, (**b**) with 7.5 μM penfluridol for the indicated time points. **c** U937 and HL-60 cells were pretreated with and without 20 μM U0126, SP600125, or 1 μM SB203580 for 1 h followed by 7.5 μM penfluridol treatment for an additional 8 h. Expression levels of cleaved poly (ADP ribose) polymerase (PARP) and phosphorylation levels of ERK1/2, JNK1/2, and p38 were determined by a Western blot analysis. **d** HL-60, U937, and MV4–11 cells were treated with penfluridol (5 or 7.5 μM) or FTY720 (5 μM) for 24 h and phosphorylation levels of PP2A-Cα were determined by a Western blot analysis. **e** and **f** Pretreatment of MV4–11 and HL-60 cells with different concentration of okadaic acid (OA) for 1 h followed by 7.5 μM penfluridol treatment for an additional 8 h and the phosphorylation levels of ERK and JNK (**e**), and cleaved caspase-3 (**f**) were determined by a Western blot analysis. **g** Kaplan-Meier plots for high versus low PP2A-C RNA expression in AML. A low PP2A-C RNA expression level was correlated with a poor prognosis in RNA sequencing analysis from the Gene Expression Omnibus (GEO) database (GSE10358) (*p* = 0.026). **h** Survival analysis of RNA microarray or RNA sequencing data from the SurvExpress database was assessed in six cohorts, results showed that PP2A-C is a significant prognostic predictive indicator for AML, and patients with high PP2A-C expression had favorable prognoses. The horizontal lines indicate the confidence intervals (CIs)

Previous studies indicated that the PI3K/Akt and MAPK pathways, such as ERK and JNK, are important downstream proliferative signaling pathways of FLT3 [26, 27]. Therefore, we determined whether Akt and MAPK activation was affected by penfluridol treatment of AML cells, and found that penfluridol suppressed activation of Akt, JNK, and ERK1/2, but induced p38 MAPK activation at different time points in U937 and HL-60 cells (Fig. 3a). Similar results were also observed in penfluridol-treated MV4–11 cells (Fig. 3b). We next examined whether the MAPK pathways were involved in penfluridol-induced apoptosis of AML cells. HL-60 and U937 cells were pretreated with 20 μM U0126 (an ERK inhibitor), SP600125 (a JNK inhibitor), or 1 μM SB203580 (a p38 inhibitor) for 1 h, treated with 7.5 μM penfluridol for another 8 h, and then analyzed by Western blotting. We found that penfluridol-induced cleavage of PARP was enhanced by U0126 and SP600125, but was rescued by SB203580 (Fig. 3c). A recent study showed that pharmacological activation of the serine/threonine phosphatase, PP2A, suppresses FLT3-mediated growth of AML cells [11]. Moreover, PP2A was demonstrated to act as a deactivator of several kinases, such as MAPKs and Akt [28]. Hence, we hypothesized that penfluridol can activate PP2A to deactivate MAPKs and Akt and subsequently suppress AML growth. Previous reports indicated that phosphorylation of the PP2A-C [29, 30] at the Tyr 307 site can attenuate PP2A activity. We found that treatment of penfluridol or PP2A activator, FTY720, with three tested AML cells all can attenuate the phosphorylation status of PP2A-Cα, suggesting PP2A activity is actually increased in penfluridol-treated AML cells (Fig. 3d). Moreover, we noted that pretreatment of PP2A inhibitor, okadaic acid, can rescue penfluridol-induced deactivation of ERK and JNK, and activation of caspase-3 in both HL-60 and MV4–11 cells (Fig. 3e and f), suggesting that the penfluridol-induced apoptotic effect in AML cells might be through induction of PP2A activation to negatively regulate ERK and JNK activities. In the clinic, we analyzed the prognostic significance of PP2A-C expression using gene expression data obtained from the publicly available Gene Expression Omnibus (GEO) database (GSE10358). Results from a Kaplan-Meier plot showed that patients with AML that exhibited high expression levels of PP2A-C had significantly longer overall survival times (*p* = 0.026) compared to patients with AML that exhibited low expression levels of PP2A-C (Fig. 3g). Moreover, results of a survival analysis of RNA microarray or RNA sequencing data from the SurvExpress web resource (http://bioinformatica.mty.itesm.mx/SurvExpress) of six other cohorts showed that PP2A-C was a significant prognostic predictive indicator for AML, and patients with high PP2A-C expression had favorable prognoses (Fig. 3h).

### Increased intracellular oxidative stress plays a protective role in the penfluridol-induced apoptotic effect

**Fig. 4:**
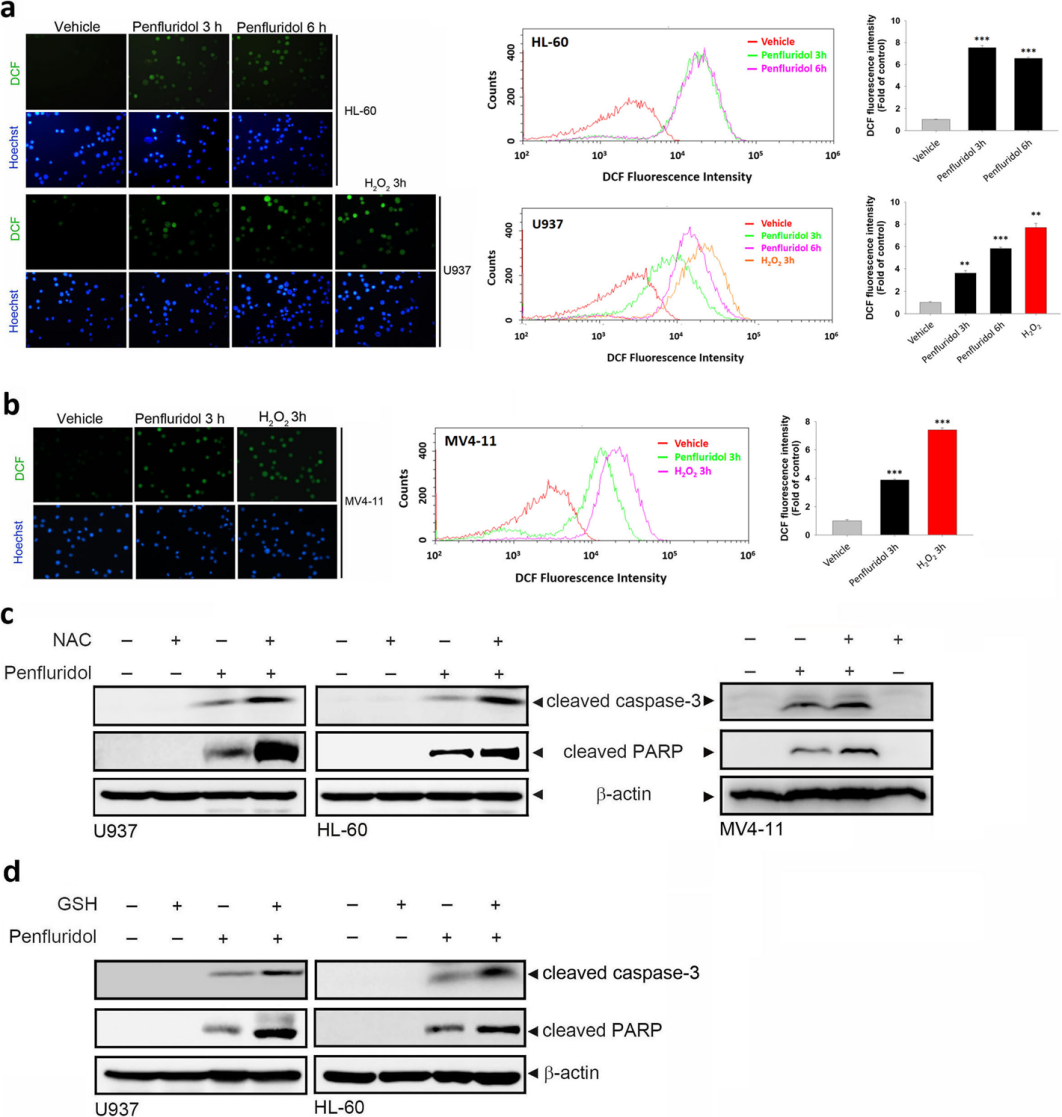
Increased intracellular oxidative stress plays a protective role in penfluridol-induced apoptosis of acute myeloid leukemia (AML) cells. **a** and **b** Fms-like tyrosine kinase 3 (FLT3)-wild type (WT) AML cells, U937 and HL-60, (**a**) or FLT3 mutant (internal tandem duplication) cells, MV4–11, (**b**) were treated with 7.5 μM penfluridol or 100 μM H_2_O_2_ for the indicated time, and then the reactive oxygen species (ROS) level was measured by H_2_DCFDA staining under a fluorescence microscope (left panel) or flow cytometric analysis (right panel). Original magnification, 200×. Data are presented as the mean ± SD. ** *p* < 0.01; *** *p* < 0.001 versus the vehicle control group. **c** and **d** U937, HL-60, and MV4–11 cells were pretreated with and without 5 mM N-acetylcysteine (NAC) (**c**) or glutathione (GSH) (**d**) for 1 h followed by treatment with 7.5 μM (U937 and HL-60) or 5 μM (MV4–11) penfluridol for 24 h; cleaved PARP and caspase-3 were detected by a Western blot analysis. β-actin served as a loading control

In addition to the PP2A/MAPK pathway, penfluridol was shown to induce cell apoptosis of breast cancer via inducing ROS production [19]. Thus, we examined whether penfluridol-induced apoptosis involved the generation of ROS in AML cells. Cellular ROS were monitored with the fluorescent, redox-sensitive dye, H_2_DCFDA, after treatment of U937 or HL-60 cells with penfluridol. H_2_DCFDA is widely used to measure cellular H_2_O_2_, so H_2_O_2_ was used as a positive control. The fluorescence microscopic (Fig. 4a, left panel) and flow cytometric (Fig. 4a, right panel) analyses showed that penfluridol and H_2_O_2_ induced an increase in DCF green fluorescence compared to the vehicle-treated group. ROS production induced by penfluridol was also observed in MV4–11 cells (Fig. 4b). Surprisingly, we found that pretreating U937, HL-60, or MV4–11 cells with the antioxidant, NAC, enhanced penfluridol-induced caspase-3 activation and PARP cleavage (Fig. 4c). A similar enhanced effect on penfluridol-induced caspase-3 activation and PARP cleavage was induced by another antioxidant, GSH (Fig. 4d), suggesting that ROS production might play a protective role in the penfluridol-induced apoptotic effect in AML cells harboring FLT3-WT and FLT3-ITD.

### Penfluridol induces ROS-mediated autophagy via triggering LC3 turnover and AVO formation

**Fig. 5:**
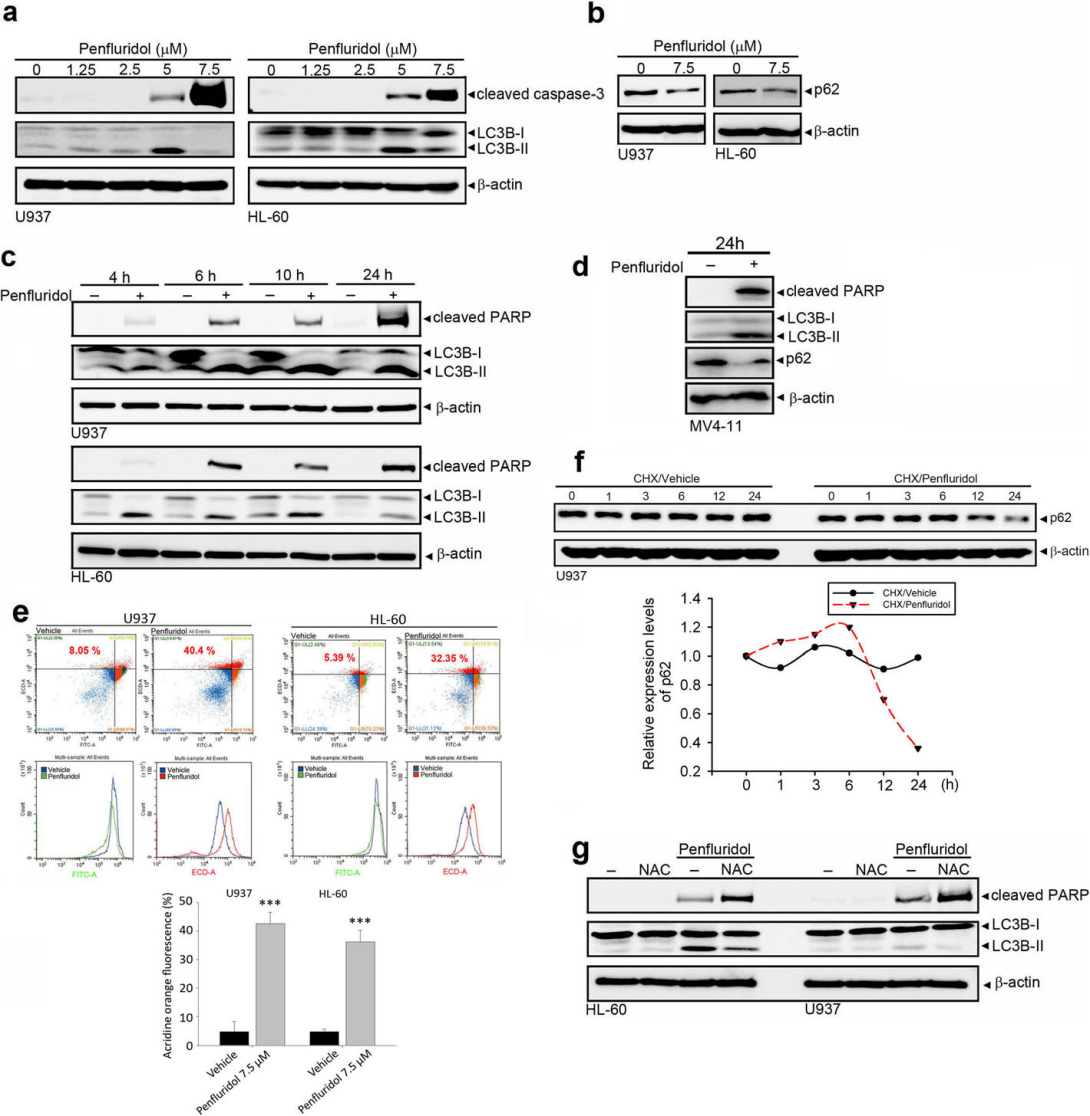
Penfluridol induces reactive oxygen species (ROS)-mediated autophagy via triggering light chain 3 (LC3) turnover and acidic vesicular organelle (AVO) formation. **a** and **b** U937 and HL-60 cells were treated with the indicated concentrations of penfluridol for 24 h. Cell lysates were probed with antibodies against cleaved caspase-3, LC3 (**a**), and p62 (**b**). **c** and **d** Treatment of U937 and HL-60 cells with 7.5 μM penfluridol (**c**) or treatment of MV4–11 cells with 5 μM penfluridol (**d**) for the indicated time points. LC3 conversion (LC3-I to LC3-II), pro-poly (ADP ribose) polymerase (PARP) cleavage, and p62 degradation were detected by a Western blot analysis. β-actin served as a loading control. **e** U937 and HL-60 cells treated with 7.5 μM penfluridol for 12 h were stained with acridine orange (AO) and subjected to flow cytometry. Top of the grid was considered AVOs. Data are presented as the mean ± SD. *** *p* < 0.001 versus the vehicle control group. **f** U937 cells were treated with 35 μM cycloheximide or cycloheximide plus penfluridol for the indicated time points. The p62 degradation was detected by a Western blot analysis. Quantitative results of p62 protein levels are shown at the bottom. **g** U937 and HL-60 cells were exposed to N-acetylcysteine (NAC) (5 mM) for 1 h, followed by treatment with penfluridol (7.5 μM) for 24 h, and levels of LC3 conversion and cleaved PARP were analyzed by Western blotting. β-actin was the internal standard for protein loading

Autophagy is type II programmed cell death and currently reported to be induced by penfluridol in solid tumors [22]. Hence, we further checked if autophagy had any role in the death of leukemia cells induced by penfluridol. LC3 turnover (LC3B-I to LC3B-II conversion) and p62 degradation are hallmarks of autophagy, and Western blotting was used to assay the effect of penfluridol on LC3 turnover and p62 protein levels. As shown in Fig. 5a, 5 μM and 7.5 μM penfluridol respectively induced dominant LC3B-II formation and caspase-3 activation in U937 and HL-60 cells. p62 degradation induced by penfluridol was also observed in both AML cell lines (Fig. 5b). According to data from different time points (4, 6, 10, and 24 h) of penfluridol treatment of U937 and HL-60 cells, we found that penfluridol induced PARP cleavage and LC3 turnover beginning at 4 h, and these effects were maintained for 24 h after treatment (Fig. 5c), indicating that autophagy and apoptosis might simultaneously be induced by penfluridol. In addition to FLT3-WT AML cells, 24-h treatment of penfluridol also simultaneously induced PARP cleavage and LC3 turnover as well as p62 degradation in MV4–11 cells (Fig. 5d). To further examine the effect of penfluridol on autophagic flux, we detected the formation of AVOs by flow cytometry using the lysosomotropic agent, AO staining. We observed that significant formation of AVOs was induced by penfluridol treatment in U937 and HL-60 cells compared to vehicle-treated cells (Fig. 5e). Moreover, cellular level of p62 was negatively correlated with autophagic flux [31]. We observed that penfluridol can induce the degradation of p62 and decrease the half-life of p62 in cycloheximide (CHX)-treated U937 cells (Fig. 5f), suggesting penfluridol can promote the autophagic flux in AML cells. We next investigated the significance of ROS production in penfluridol-triggered autophagy and apoptosis in AML cells. Pretreatment with NAC enhanced penfluridol-induced PARP cleavage and attenuated penfluridol-induced LC3 turnover and p62 degradation (Fig. 5g, Additional file 1: Figure S1), suggesting that penfluridol can induce production of ROS which led to prevention of apoptosis and induction of autophagy.

### Penfluridol induces caspase-mediated crosstalk between apoptosis and autophagy

**Fig. 6:**
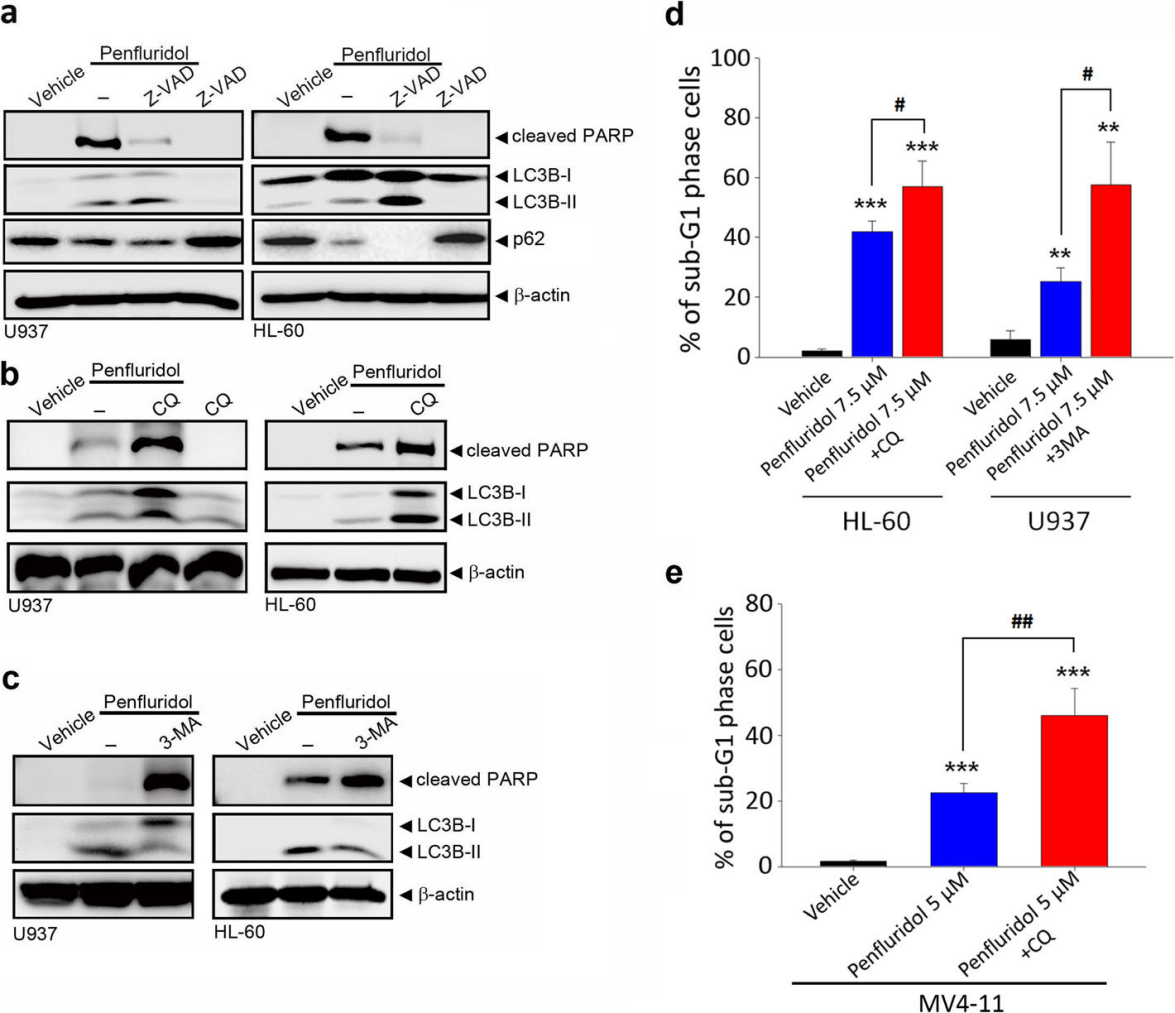
Autophagy inhibition enhances the penfluridol-induced apoptotic effect in acute myeloid leukemia (AML) cells. **a-c** U937 and HL-60 cells were pretreated with 20 μM Z-VAD-fmk (**a**), chloroquine (CQ) (**b**), or 10 mM 3-methylamphetamine (3-MA) (**c**) for 1 h followed by penfluridol (7.5 μM) treatment for an additional 24 h. Levels of cleaved pro-poly (ADP ribose) polymerase (PARP), light chain (LC)3 conversion, and p62 degradation were detected by a Western blot analysis, and β-actin was used as a loading control. **d** and **e** U937, HL-60, and MV4–11 cells were pretreated with 20 μM CQ or 10 mM 3-MA for 1 h followed by 7.5 μM (**d**) or 5 μM (**e**) penfluridol treatment for an additional 24 h. The sub-G_1_ cell population was analyzed by flow cytometry following staining with propidium iodide (PI). Data are presented as the mean ± SD. ** *p* < 0.01; *** *p* < 0.001 versus the vehicle control group. ^#^ *p* < 0.05; ^##^ *p* < 0.01 versus the penfluridol treatment only group

To investigate crosstalk between autophagy and apoptosis induced by penfluridol in AML cells, the pan-caspase inhibitor, Z-VAD-fmk, the early autophagy inhibitor, 3-MA, and the late autophagy inhibitor, CQ, were used. As expected, pretreatment of HL-60 and U937 cells with Z-VAD-fmk significantly reversed penfluridol-induced PARP cleavage. In contrast, Z-VAD-fmk pretreatment promoted penfluridol-induced LC3 turnover and p62 degradation (Fig. 6a), suggesting that caspase activation is also involved in modulating penfluridol-induced cell autophagy. Moreover, regardless of whether AML cells were pretreated with CQ or 3-MA, both dramatically enhanced penfluridol-induced PARP cleavage (Fig. 6b, c), compared to that of penfluridol treatment only. Furthermore, higher sub-G_1_ cell accumulations induced by penfluridol combined with CQ or 3-MA than the penfluridol-only treatment group were observed in U937, HL-60, and MV4–11 cells (Fig. 6d, e), suggesting that the apoptotic effect induced by penfluridol was augmented by autophagy inhibition in AML cells harboring FLT3-WT and FLT3-ITD.

## Discussion

Inactivation of the tumor suppressor, PP2A, frequently occurs in AML, leading to sustained activation of survival pathways or inhibition of apoptotic pathways, and is a critical recurrent event in AML [9, 10]. Oncogenic FLT3 activation was recently reported to inactivate PP2A in FLT3-WT and FLT3-ITD AML cell lines or primary human AML blasts. AML blasts from FLT3-ITD patients displayed lower PP2A activity than FLT3-WT blasts [11]. The main finding of the present study is that penfluridol, an antipsychotic drug, exerted an anticancer effect on AML cells harboring FLT3-WT and FLT3-ITD through activating PP2A to deactivate downstream Akt, ERK, and JNK signals and subsequently triggered caspase-mediated cellular apoptosis. We also observed that penfluridol exhibited more-potent cytotoxicity against FLT3-ITD AML cells (MV4–11) than FLT3-WT AML cells (HL-60 and U937), which might have been due to FLT3-ITD cells displaying lower PP2A activity than FLT3-WT cells [11]. Indeed, previous studies showed that leukemic cells with low PP2A activity were sensitive to cell death induced by the PP2A-activating drug (PAD), FTY720 [32], and FLT3-ITD cells were more sensitive to inhibition of proliferation by FTY720 than were FLT3-WT AML cells [11].

In contrast to suppression of ERK and JNK activation by penfluridol, p38 MAPK activation induced by penfluridol was also observed in AML cells. It was reported that p38 activation negatively regulates ERK activity in a PP2A-dependent manner and further induces apoptosis of cardiac myocytes [33]. In colorectal cancer, the PP2A-C level was demonstrated to determine their differential responses to a p38 inhibitor. PP2A-C was expressed at a much higher level in the p38 inhibitor-induced survival group than in p38 inhibitor-induced apoptosis group. This phenomenon was due to the negative regulation of p38 on ERK through PP2A [34]. Our current study showed that penfluridol-induced ERK dephosphorylation and PARP cleavage were significantly reversed by the p38 inhibitor, SB203580 (Additional file 1: Figure S2), suggesting that p38 activation can negatively regulate ERK activity to induce apoptosis in penfluridol-treated AML cells. The role of PP2A in p38-regulated ERK dephosphorylation in penfluridol-treated AML cells will be further investigated in the future.

The current therapeutic decision for AML treatment consists of cytotoxic chemotherapy and tyrosine kinase inhibitors (TKIs) [35]. However, chemotherapeutic treatments are often accompanied by elevated ROS levels, which cause drug resistance due to ROS-mediated prosurvival pathways including RAS/MAPK cascades and PI3K/Akt pathways [36, 37] . ROS were also shown to promote chemoresistance via inactivating PP2A (nitration of PP2A-B56δ) and leading to increased phosphorylation of S70 Bcl-2 in hematopoietic malignancies [38]. Our study showed that penfluridol can induce activation of PP2A and deactivation of Akt and MAPKs (ERK and JNK), suggesting that penfluridol might be a potential tool to overcome chemoresistance in AML. However, similar to chemotherapeutic drugs, penfluridol treatment also triggered an ROS-mediated prosurvival effect in AML cells. Moreover, inhibition of ROS by two antioxidants, NAC and GSH, further enhanced the apoptosis-inducing effect of penfluridol in AML cells. Similar results from previous clinical trials, which examined the effects of dietary antioxidants taken concurrently with chemotherapy, found that GSH combined with cisplatin-based chemotherapy could improve the antitumor response against leukemia [39]. Therefore, combinations of penfluridol, antioxidants, and chemotherapeutic agents might have promising synergistic effects in AML.

In addition to apoptotic cell death induced by penfluridol, autophagy induction was also observed in penfluridol-treated AML cells. The role of autophagy in AML progression is highly controversial. Interestingly, induction of autophagy was reported to suppress tumorigenesis, whereas autophagy induction is also known to support tumor growth and chemoresistance in AML [40]. Our current study showed that pretreatment with the pan-caspase inhibitor, Z-VAD-fmk, respectively prevented and promoted penfluridol-induced apoptosis and autophagy. Moreover, inhibition of autophagy by an early and a late autophagy inhibitor both enhanced penfluridol-induced apoptotic cell death of AML cells. These results indicated that penfluridol-mediated autophagy is a prosurvival mechanism rather than a pro-death mechanism, and caspase activation might negatively regulate autophagy in penfluridol-treated cells. The interplay between caspases and autophagy-related proteins (ATGs) in modulating autophagic flux was previously reported by several review articles [41], but the crosstalk between caspases and ATG regulated by penfluridol needs to be further investigated in the future. Recently, various anticancer drugs were shown to activate ROS-mediated autophagy simultaneously leading to cytoprotective regulation and induction of cellular apoptosis [42, 43]. Results described herein indicated that autophagy induction by penfluridol is also dependent on ROS generation as evidenced by the rescue of penfluridol-induced LC3 turnover by NAC. Taken together, these findings indicated that ROS-mediated autophagy provides a cytoprotective mechanism in human AML cells treated with penfluridol, and inhibition of ROS-mediated autophagy may improve the therapeutic efficacy of penfluridol in AML treatment.

## Conclusions

**Fig. 7:**
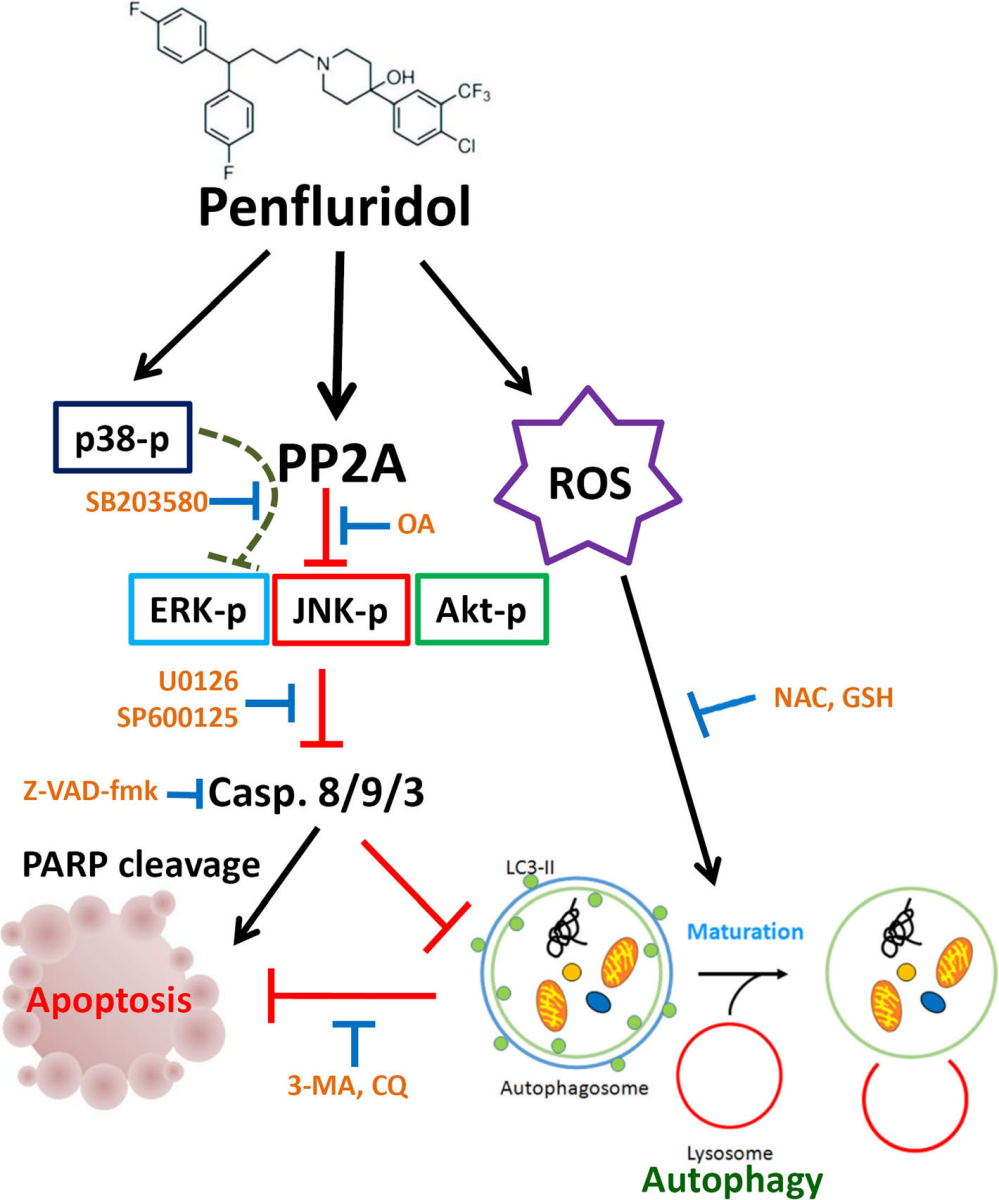
A working model shows the molecular mechanism underlying the ability of penfluridol to induce apoptotic cell death of acute myeloid leukemia (AML) cells. The antileukemic effect of penfluridol was attributed to its induction of caspase-mediated apoptosis via protein phosphatase 2A (PP2A)-mediated deactivation of Akt, extracellular signal-regulated kinase (ERK), and c-Jun N-terminal kinase (JNK). Meanwhile, penfluridol not only triggers apoptosis but also induces ROS-mediated autophagy. The ROS-mediated autophagy induced by penfluridol may have been due to a cell-derived protective effect against the toxic effects of penfluridol. Bold dashed lines indicate hypothetical pathways which might be regulated by penfluridol

The present study demonstrates for the first time, that treatment of AML cells harboring FLT3-WT or FLT3-ITD with penfluridol induces obvious apoptosis through inducing PP2A-mediated deactivation of Akt, ERK, and JNK signaling. Moreover, apoptosis is not the only consequence of penfluridol treatment, as penfluridol treatment simultaneously activates ROS-mediated cytoprotective autophagy. Inhibition of ROS-mediated autophagy significantly enhanced penfluridol-induced apoptotic cell death. The schematic mechanism is illustrated in Fig. 7 and indicates that AML cell apoptosis and autophagy elicited by penfluridol are modulated by caspase activation. Recently, several preclinical studies reported that the pharmacological restoration of PP2A tumor-suppressor activity by PADs effectively antagonizes leukemogenesis, and the anticancer activity of PADs usually depends on interactions with PP2A’s endogenous inhibitor, SET, an oncogene overexpressed in 28% of AML patients [9, 44, 45]. Although whether penfluridol can prevent SET/PP2A-C binding needs be further addressed, our present findings strongly support repurposing penfluridol as a drug for treating AML, especially for the cancer types that are characterized by functional loss of the PP2A tumor suppressor.

## Additional file

Additional file 1: **Figure S1.** Inhibition of reactive oxygen species (ROS) reverses penfluridol-induced LC3 turnover and p62 degradation in HL-60 acute myeloid leukemia cells. **Figure S2.** Inhibition of p38 mitogen-activated protein kinase (MAPK) reverses penfluridol-induced extracellular signal-regulated kinase (ERK) dephosphorylation in U937 and HL-60 acute myeloid leukemia cells. (DOCX 166 kb)

## Data Availability

All data used during the current study are available from the corresponding author on reasonable request.

## Abbreviations

3-MA: 3-methylamphetamine
AML: acute myeloid leukemia
ATGs: autophagy-related proteins
AVO: acidic vesicular organelle
CQ: chloroquine
ERK: extracellular signal-regulated kinase
FLT3: Fms-like tyrosine kinase 3
GSH: glutathione
ITD: internal tandem duplication
JNK: c-Jin N-terminal kinase
LC3: light chain 3
MAPK: mitogen-activated protein kinase
NAC: N-acetylcysteine
OA: okadadic acid
PADs: PP2A-activating drugs
PARP: poly (ADP ribose) polymerase
PP2A: protein phosphatase 2A
ROS: reactive oxygen species
TKI: tyrosine kinase inhibitor
WT: wild type
