# Supplementary material for: Penfluridol triggers cytoprotective autophagy and cellular apoptosis through ROS induction and activation of the PP2A-modulated MAPK pathway in acute myeloid leukemia with different FLT3 statuses
Source: J Biomed Sci. 2019 Aug 31;26:63. doi: 10.1186/s12929-019-0557-2 (PMC6717358; doi:10.1186/s12929-019-0557-2)
Supplement: Supplementary file 1 — Figure S1. Inhibition of reactive oxygen species (ROS) reverses penfluridol-induced LC3 turnover and p62 degradation in HL-60 acute myeloid leukemia cells. Figure S2. Inhibition of p38 mitogen-activated protein kinase (MAPK) reverses penfluridol-induced extracellular signal-regulated kinase (ERK) dephosphorylation in U937 and HL-60 acute myeloid leukemia cells. (DOCX 166 kb) [file 12929_2019_557_MOESM1_ESM.docx]

**Supplemental Information**

**Title:**

**Penfluridol triggers cytoprotective autophagy and cellular apoptosis through ROS induction and the PP2A-modulated MAPK pathway in acute myeloid leukemia with different FLT3 statuses**

Szu‐Yuan Wu, Yu-Ching Wen, Chia-Chi Ku, Yi-Chieh Yang, Jyh-Ming Chow, Shun-Fa Yang, Wei-Jiunn Lee, and Ming-Hsien Chien

^*^Correspondence to: Dr. Wei-Jiunn Lee (E-mail: lwj5905@gmail.com) and Dr. Ming-Hsien Chien (E-mail: mhchien1976@gmail.com)

**Figure Legend**

**
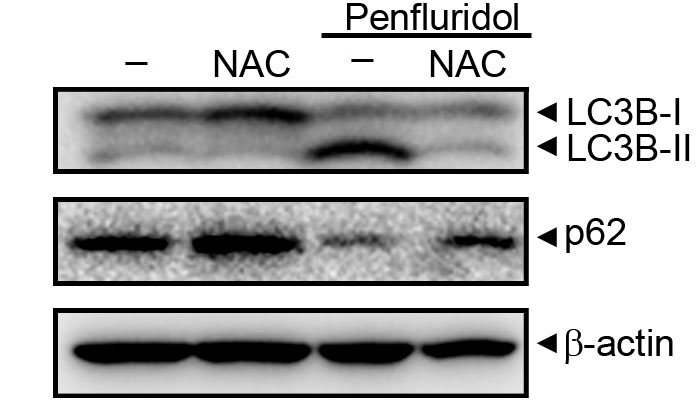
**

**Additional file 1: Figure S1.** Inhibition of reactive oxygen species (ROS) reverses penfluridol-induced LC3 turnover and p62 degradation in HL-60 acute myeloid leukemia cells. HL-60 cells were pretreated with and without 5 mM N-acetylcysteine (NAC) for 1 h followed by 7.5 µM penfluridol treatment for 24 h. Expression levels of LC3 and p62 were determined by a Western blot analysis, and β-actin served as a loading control.

**
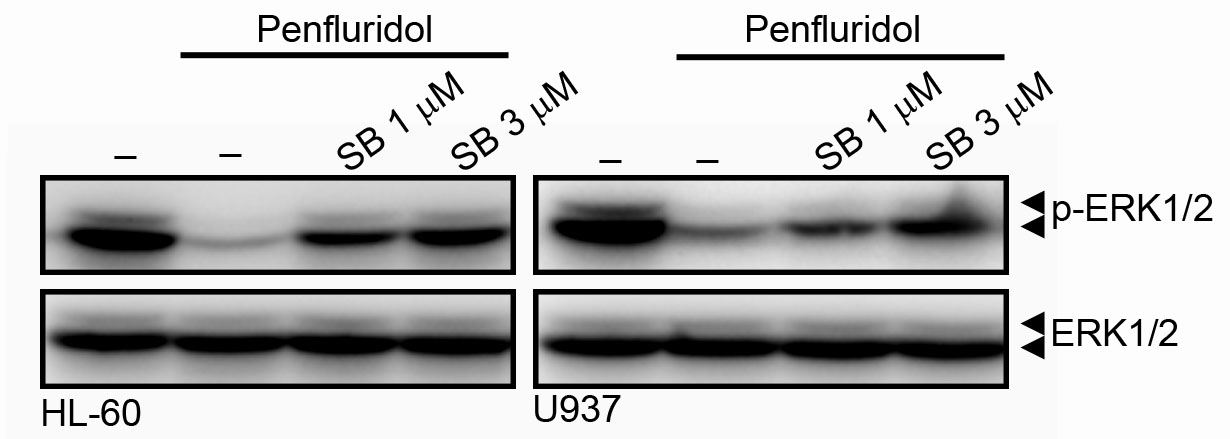
**

**Additional file 1: Figure S2.** Inhibition of p38 mitogen-activated protein kinase (MAPK) reverses penfluridol-induced extracellular signal-regulated kinase (ERK) dephosphorylation in U937 and HL-60 acute myeloid leukemia cells. U937 and HL-60 cells were pretreated with and without 1 or 3 μM SB203580 for 1 h followed by 7.5 µM penfluridol treatment for an additional 8 h. Phosphorylation levels of ERK1/2 were determined by a Western blot analysis, and total ERK served as a loading control.
